# Supplementary material for: The causal relationship between the human gut microbiota and pyogenic arthritis: a Mendelian randomization study
Source: Front Cell Infect Microbiol. 2024 Nov 26;14:1452480. doi: 10.3389/fcimb.2024.1452480 (PMC11629706; doi:10.3389/fcimb.2024.1452480)
Supplement: Supplementary file 1 [file DataSheet1.zip › mendelian test/ebi-a-GCST90027596.csv_forest.pdf]

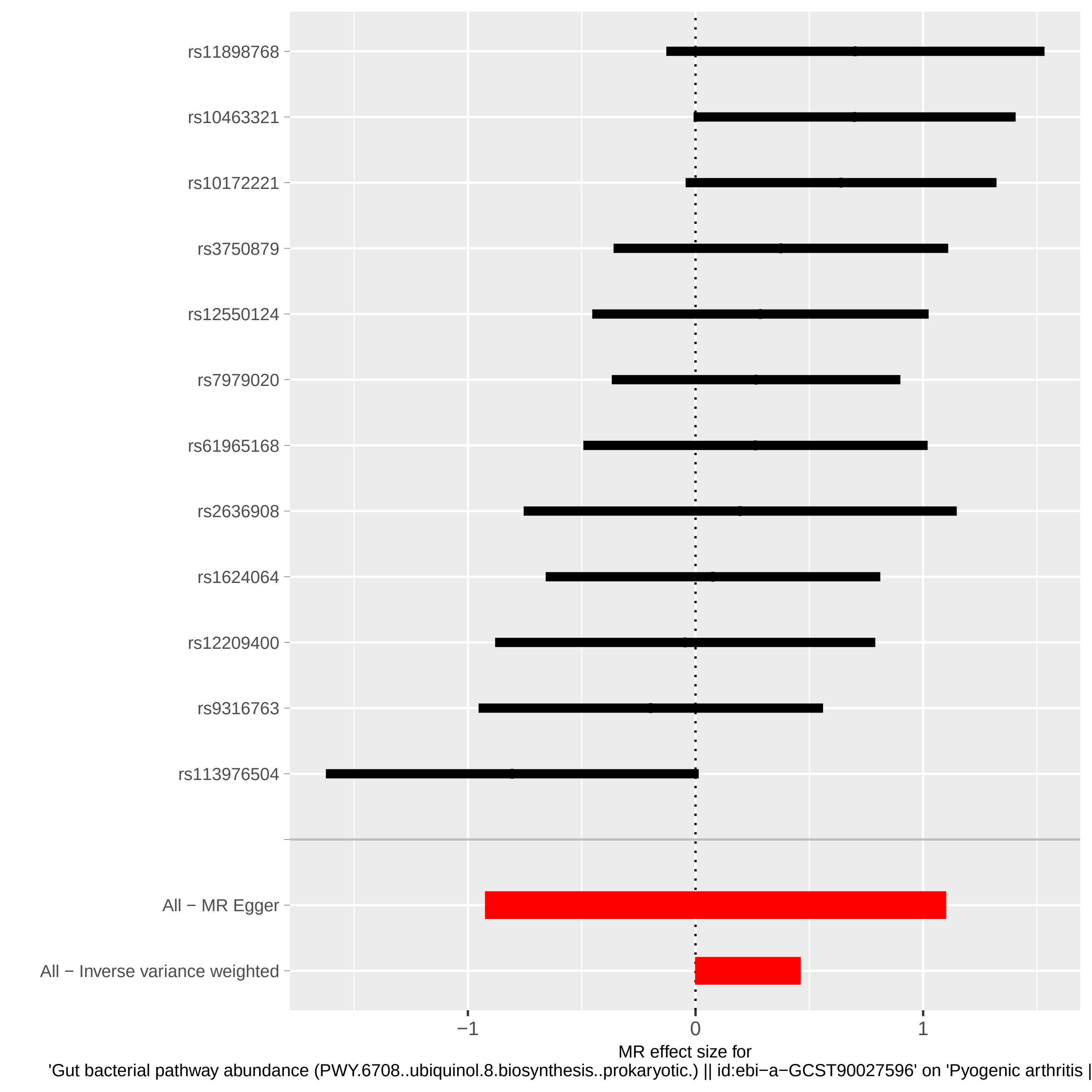
rs11898768
rs10463321
rs10172221
rs3750879
rs12550124
rs7979020
rs61965168
rs2636908
rs1624064
rs12209400
rs9316763
rs113976504
All − MR Egger
All − Inverse variance weighted
−1
0
1
MR effect size for
'Gut bacterial pathway abundance (PWY.6708..ubiquinol.8.biosynthesis..prokaryotic.) || id:ebi−a−GCST90027596' on 'Pyogenic arthritis |
